# Propylene/propane permeation properties of ethyl cellulose (EC) mixed matrix membranes fabricated by incorporation of nanoporous graphene nanosheets

**DOI:** 10.1038/srep28509

**Published:** 2016-06-29

**Authors:** Bingbing Yuan, Haixiang Sun, Tao Wang, Yanyan Xu, Peng Li, Ying Kong, Q. Jason Niu

**Affiliations:** 1 State Key Laboratory of Heavy Oil Processing, China University of Petroleum (East China), Qingdao 266580, P.R. China; 2 College of Science, China University of Petroleum (East China), Qingdao 266580, P.R. China

## Abstract

Nanopore containing graphene nanosheets were synthesized by graphene oxide and a reducing agent using a facile hydrothermal treatment in sodium hydroxide media. The as-prepared nanoporous graphene was incorporated into ethyl cellulose (EC) to prepare the mixed matrix membranes (MMMs) for C_3_H_6_/C_3_H_8_ separation. Transmission electron microscopy (TEM) photograph and X-ray photoelectron spectroscopy (XPS) analysis of nanoporous graphene nanosheets indicated that the structure of nano-pore was irregular and the oxygen-containing groups in the surface were limited. More importantly, the as-prepared MMMs presented better separation performance than that of pristine EC membrane due to simultaneous enhancement of C_3_H_6_ permeability and ideal selectivity. The ideal selectivity of the MMMs with 1.125 wt‰ nanoporous graphene content for C_3_H_6_/C_3_H_8_ increased from 3.45 to 10.42 and the permeability of C_3_H_6_ increased from 57.9 Barrer to 89.95 Barrer as compared with the pristine membrane. The presumed facilitated mechanism was that the high specific surface area of nanoporous graphene in polymer matrix increased the length of the tortuous pathway formed by nanopores for the gas diffusion as compared with the pristine graphene nanosheets, and generated a rigidified interface between the EC chains and fillers, thus enhanced the diffusivity selectivity. Therefore, it is expected that nanoporous graphene would be effective material for the C_3_H_6_/C_3_H_8_ separation.

The separation of low-carbon olefin/paraffin mixtures is one of the most challenging tasks in the petrochemical industry due to their similar molecular sizes and physical properties. Currently, the separation is mainly achieved by fractional distillation at cryogenic temperature^1^, and large capital investment and high energy consumption involved in this conventional technology stimulates the researchers to find a more cost-effective separation process^2^. As an alternatively energy-saving approach, membrane separation technology has a great potential in the petrochemical industry^3^,^4^,^5^. Mixed matrix membranes (MMMs) that are composed by blending inorganic particles into the polymer matrix are promising approaches; they combine the easy processing polymeric membranes with superior separation performance of inorganic nanoparticles^6^. These advantages also provide an opportunity to overcome the individual deficiencies of inorganic materials and polymers, offering attractive solutions for industrial applications. Correspondingly, there are several inorganic materials such as zeolites, carbon molecular sieves (CMS), carbon nanotubes (CNTs), C_60_, metal-organic frameworks (MOFs), and covalent organic frameworks (COFs) that have been amalgamated into the polymer matrix to prepare the MMMs for gas separation, and subsequently achieved enhanced CO_2_/CH_4_, O_2_/N_2_, H_2_/CO_2_ and CO_2_/N_2_ selectivity^7^,^8^,^9^,^10^,^11^,^12^.

In recent years, a few studies have been reported about employing mixed matrix platform to the C_3_H_6_/C_3_H_8_ system using nanofillers, and most nanofillers are spherical shape like silica materials and C_60_, or square zeolitic like imidazolate framework ZIF-8 13,14,15. For example, Naghsh and Sadeghi^14^ studied the separation of propylene/propane using cellulose acetate-silica nanocomposite membranes, and the selectivity for C_3_H_6_/C_3_H_8_ was 6.12 and the permeability of C_3_H_6_ was 0.098 Barrer with a nanocomposite membrane that contains 30 wt% silica particles under 2 bar feed absolute pressure and 35 °C temperature. Koros *et al*.^13^ found that mixed matrix membranes, fabricated by 6FDA-DAM polyimide and ZIF-8, had an ideal selectivity of 31.0 for C_3_H_6_/C_3_H_8_, and a permeability of 56.2 Barrer for C_3_H_6_ with 48.0 wt% ZIF-8 loading, which were 150% and 258% higher than the pure 6FDA-DAM membrane respectively for selectivity and permeability.

As mentioned above, most available literatures focused on the spherical and square shape nanofillers to enhance the gas separation performance. It is inevitable to generate the ‘sieve-in-a-cage morphology’ between these nanofiller materials and polymer interface due to the low aspect ratio of the spherical and square shape nanofillers and the weak interfacial adhesion. Such voids decrease the selectivity of the MMMs, even the permeability is increased^7^,^16^. In contract, the graphene nanosheets are intrinsically more compatible with polymers as compared to other quadrate or sphere molecule sieves because of its high aspect ratio (>1000), easy surface functionalization, and high thermal and mechanical properties^17^,^18^. Moreover, the high specific surface area fillers in the polymer matrix increase the length of the tortuous pathway for gas diffusion and reduce the mobility of polymer chains. This advantage will restrict the diffusion of larger molecules, and favor the diffusion of small molecules with less resistance, thus improving gas diffusivity selectivity^19^,^20^,^21^,^22^,^23^,^24^. On the other hand, Checchetto *et al*.^25^ studied the gas transport performance of nanocomposite membrane that was composed of polyethylene with dispersed graphite nanoplatelets (GNPS), and the results indicated that GNPS with nominal content of 5 wt% inclusions reduced the permeability by approximately a factor of two as compared with that of pure polymer membrane. This was due to the fact that the defect-free graphene and its derivatives like graphene oxide were theoretically gas impermeable, and such ultrathin two dimensional structure hindered gas diffusion, which was consistent with the theoretical compute studies that a perfect graphene was impermeable to gases even as small as He^26^,^27^. Therefore, it is useful to fabricate nanopores in the graphene nanosheets to further enhance the tortuous pathway of small gas molecule diffusion and hinder the big gas molecule diffusion in the polymer matrix.

For the reasons discussed above, ethyl cellulose (EC) is selected in this study as the polymer matrix based on its comparatively large free volume and relatively low glass transition temperatures, which might provide high gas permeability and a superb C_3_H_6_ diffusion coefficient. Furthermore, massive oxygen functional groups on the surface of graphene nanosheets generate better interfacial bonding strength with EC polymer^28^,^29^,^30^. To be specific, the nanoporous graphene was synthesized by sodium hydroxide using a facile hydrothermal treatment, and then the MMMs were fabricated with the addition of nanoporous graphene into EC polymer through solution blending method. In order to prove the nanopores in the surface of graphene nanosheets are beneficial for the enhancement of C_3_H_6_/C_3_H_8_ separation performance, light reduction graphene oxide (L-rGO) containing oxygen-containing groups such as carboxyl, hydroxyl group and reduction graphene oxide (rGO) were synthesized and incorporated into EC polymer to prepare MMMs for C_3_H_6_/C_3_H_8_ permeation. The morphology and microstructure of the as-prepared graphene material were confirmed, and the physical properties of MMMs in terms of microstructure, crystallization, tensile property and thermal stability (see Supplementary Information Table S1 and Fig. S3) were investigated as well. Moreover, for nanoporous graphene MMMs, the effects of feed pressure on the C_3_H_6_/C_3_H_8_ permeation performance were systematically examined and evaluated (see Supplementary Information Fig. S4 and Fig. S5).

## Results and Discussion

### Characterization of the L-rGO, rGO and nanoporous rGO nanosheets

**Figure 1:**
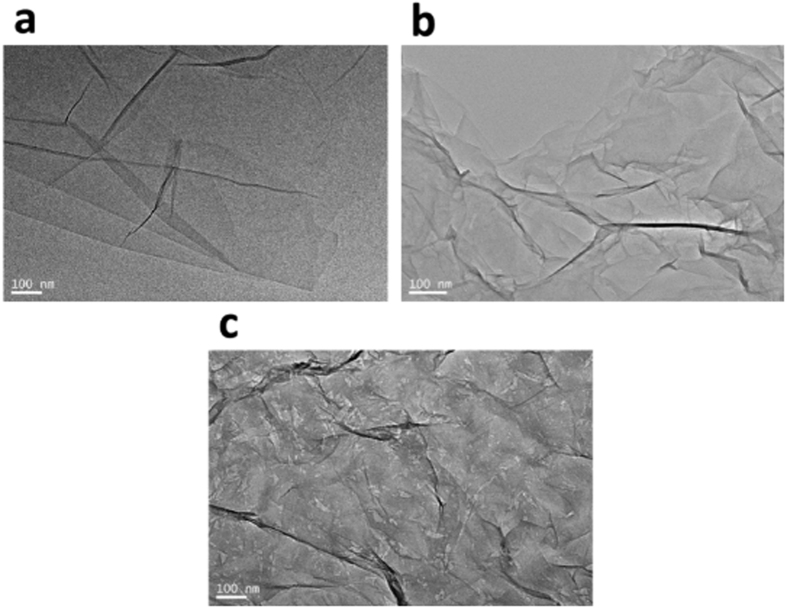
TEM images of the L-rGO nanosheets prepared under GO and NaBH_4_ mass ratio 1:2 at 80 °C for 0.5 h (**a**); the rGO nanosheets prepared under GO and NaBH_4_ mass ratio 1:25 at 80 °C for 2 h (**b**) and the nanoporous rGO nanosheets prepared using hydrothermal treatment (**c**).

Different methods of treatment on GO nanosheets may result various morphologies, and these changes can be directly observed through TEM characterization. Figure 1 shows the TEM images of L-rGO, rGO and nanoporous rGO nanosheets. As shown in Fig. 1(a), L-rGO presents relatively smooth nanosheets in comparison with rGO and nanoporous rGO nanosheets, since that trace amount of NaBH_4_ is inadequate for the chemical reduction of the vast oxygen groups on the GO nanosheets. However, intensive deoxygenation processes can be completed with abundant NaBH_4_ reduction and NaOH hydrothermal treatment, as shown in Fig. 1(b,c). The nanopores with irregular shapes are clearly distributed on the nanoporous rGO nanosheets, indicating that substantial decarbonisation and violent deoxygenation process had occurred during the hydrothermal treatment. In contrast, the rGO nanosheets practically exhibit no obvious nanopores and merely wrinkled texture, due to the deletion of oxygen groups^31^. The nanopores on the surface of rGO nanosheets may increase the specific surface area and the length of the tortuous pathway for the gas diffusion in the EC polymer matrix. In addition, the graphene nanosheets with high-aspect ratio may also contribute to the polymer and graphene nanosheets in the formation of MMMs with excellent permeability performance and mechanical properties.

**Figure 2:**
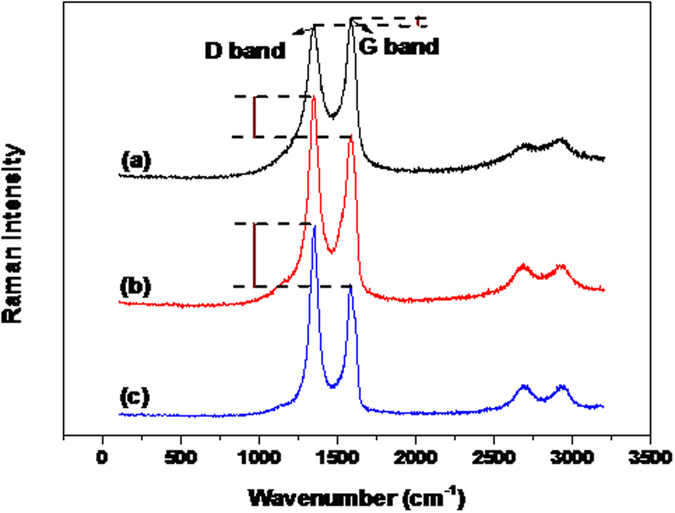
The Raman images of L-rGO nanosheets (**a**); rGO nanosheets (**b**) and nanoporous rGO nanosheets (**c**).

Variation on the morphologies of graphene nanosheets can also be indirectly measured by the Raman spectra. As observed in Fig. 2, from L-rGO to rGO, and then nanoporous rGO nanosheets, it is clearly observed that the intensity of D band gradually increases as compared with G band, and the intensity of G band marginally decreases^32^,^33^. During the chemical reduction process, the average size of sp^2^ regions in the graphene were gradually weakened and resulted in a defected surface. After that, these defected sites increasingly expanded and finally generated nanopores in the intensive hydrothermal treatment process. Therefore, an increased I_D_/I_G_ intensity ratio gradually emerges from light chemical reduction (L-rGO) to chemical reduction (rGO), and then hydrothermal treatment (nanoporous rGO). Combined with the results of TEM images, it can be concluded that nanoporous rGO nanosheets would contain large amount of nanopores as compared with L-rGO and rGO nanosheets.

**Figure 3:**
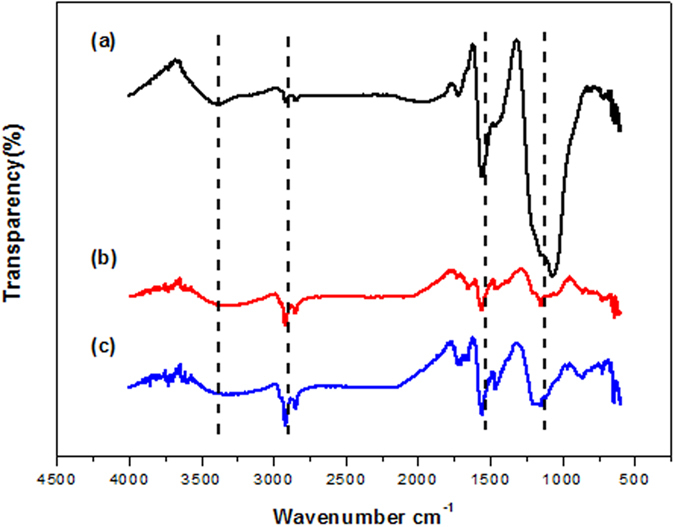
FTIR spectra of L-rGO nanosheets (**a**); rGO nanosheets (**b**) and nanoporous rGO nanosheets (**c**).

The micro-structure of graphene nanosheets were also characterized by FTIR spectroscopy. As shown in Fig. 3(a), the peaks at around 1072, 1552, and 3401 cm^−1^ wavenumber are ascribed to the C–O, C=C and O–H groups in the L-rGO with a relative higher intensity in comparison with the rGO and nanoporous rGO nanosheets^34^,^35^. On the other hand, the appearance of asymmetric bands for the alkyl groups at 2917 and 2837 cm^−1^ in rGO and nanoporous rGO results from the greatly decrease of the oxygen functional groups, indicating the completion of chemical reduction process.

**Figure 4:**
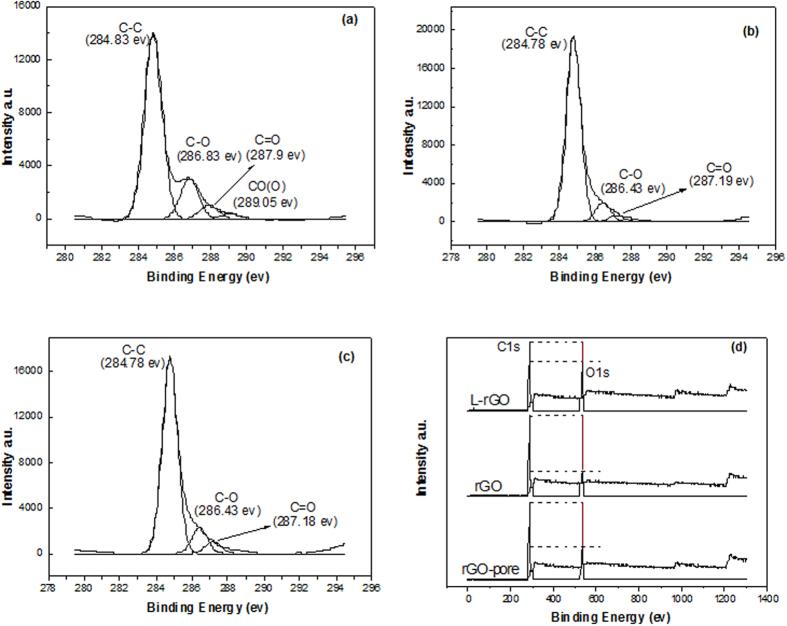
XPS spectra of L-rGO nanosheets (**a**), rGO nanosheets (**b**), nanoporous rGO nanosheets (**c**) and their XPS scan spectra (**d**).

In order to further confirm the difference of micro-structure of L-rGO, rGO and nanoporous rGO nanosheets, XPS measurement was conducted (Fig. 4). As shown in Fig. 4a, the C1s XPS spectrum of L-rGO clearly indicates a considerable degree of oxidation with four components which correspond to carbon atoms in different oxygen functional groups: the non-oxygenated ring C, the C in C–O bonds, the carbonyl C, and the carboxylate carbon (O–C=O)^36^. Moreover, L-rGO has a much lower peak intensity as compared with the C1s spectrum of rGO (Fig. 4b) and nanoporous rGO (Fig. 4c), despite possess the same oxygen functionalities. Figure 4d shows the direct atomic ratios (C1s/O1s) of L-rGO, rGO and nanoporous rGO, which also demonstrates the decrease of oxygen functionalities intensities. In addition, it is observed that rGO nanosheets exhibit lower oxygen functional intensity than that of nanoporous rGO, which are consistent with the results of FTIR.

**Figure 5:**
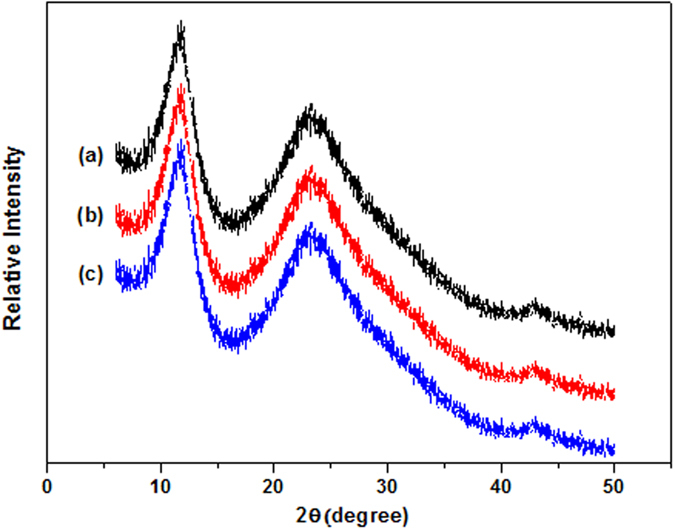
XRD patterns of L-rGO nanosheets (**a**); rGO nanosheets (**b**) and nanoporous rGO nanosheets (**c**).

Figure 5 shows the XRD patterns of L-rGO, rGO and nanoporous rGO nanosheets. The typical peak at 2θ = 11.6° is attributed to the (002) plane of GO and 2θ = 23.2°, and is the characteristic of the parallel graphene layers, indicating that L-rGO, rGO and nanoporous rGO sheets are present both the consistent layers and the size of crystallite^37^. Furthermore, morphology and micro-structure confirm that three graphene nanosheets exhibit different oxygen-containing functional groups and nanopores, that is, L-rGO nanosheets with large amounts of oxygen functional groups in the surface, rGO nanosheets with predominant aromatic rings structure in the surface, and nanoporous rGO nanosheets with aromatic rings structure and nanopores in the surface. Meanwhile, the consistent layers and the size of crystallite also show that three graphene nanosheets are no difference except oxygen functional groups and nanopores.

### Membrane Characterization

**Figure 6:**
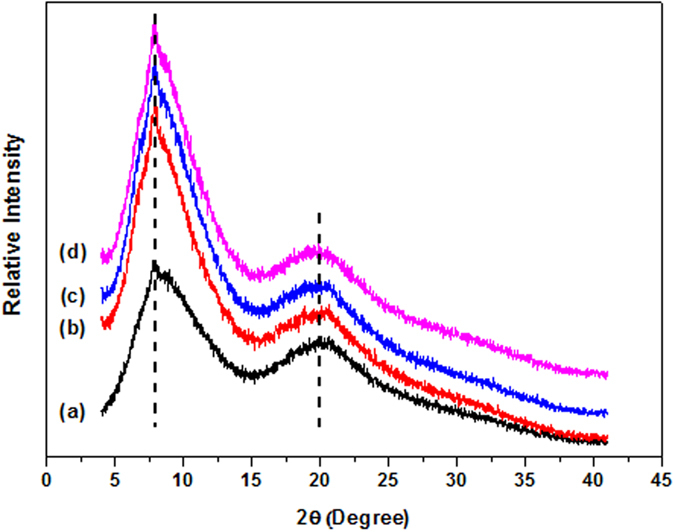
XRD patterns of pure EC membrane (**a**); L-rGO MMMs (**b**); rGO MMMs (**c**) and nanoporous rGO MMMs (**d**).

XRD is performed to investigate the effects of graphene nanosheets on the EC polymer chains. As shown in Fig. 6, the representative diffraction peaks of L-rGO, rGO and nanoporous rGO nanosheets were disappeared. Moreover, Fig. 6 also provides that the graphene nanosheets have a slight impact on the diffraction patterns of the EC polymer, especially on the variation of interlayer distance. Diffraction peaks at 2θ (°) of 7.93 and 20.56 are the characteristic of cholesteric liquid crystallinity. The small angle peak at 2θ (°) of 20.56 attributes to the interlayer distance of the ordered chains of the EC polymer, with the second peak reflecting the interchain distance^38^,^39^, therefore, graphene nanosheets have no influence on the interlayer and interchain distance of EC polymer matrix. On the other hand, the diffraction peak at 2θ (°) of 7.93 in L-rGO, rGO and nanoporous rGO nanosheets has an increased intensity as compared with that of pure EC, indicating an enhanced crystalline arrangement of EC chains. In this case, the incorporation of nanofillers into the EC polymer matrix generates a rigid interface between EC chains and graphene nanosheets, which hinders the mobility of macromolecular segments resulted from an increased chains crystallinity^40^,^41^, and eventually decreases the free volumes among polymer chains.

**Figure 7:**
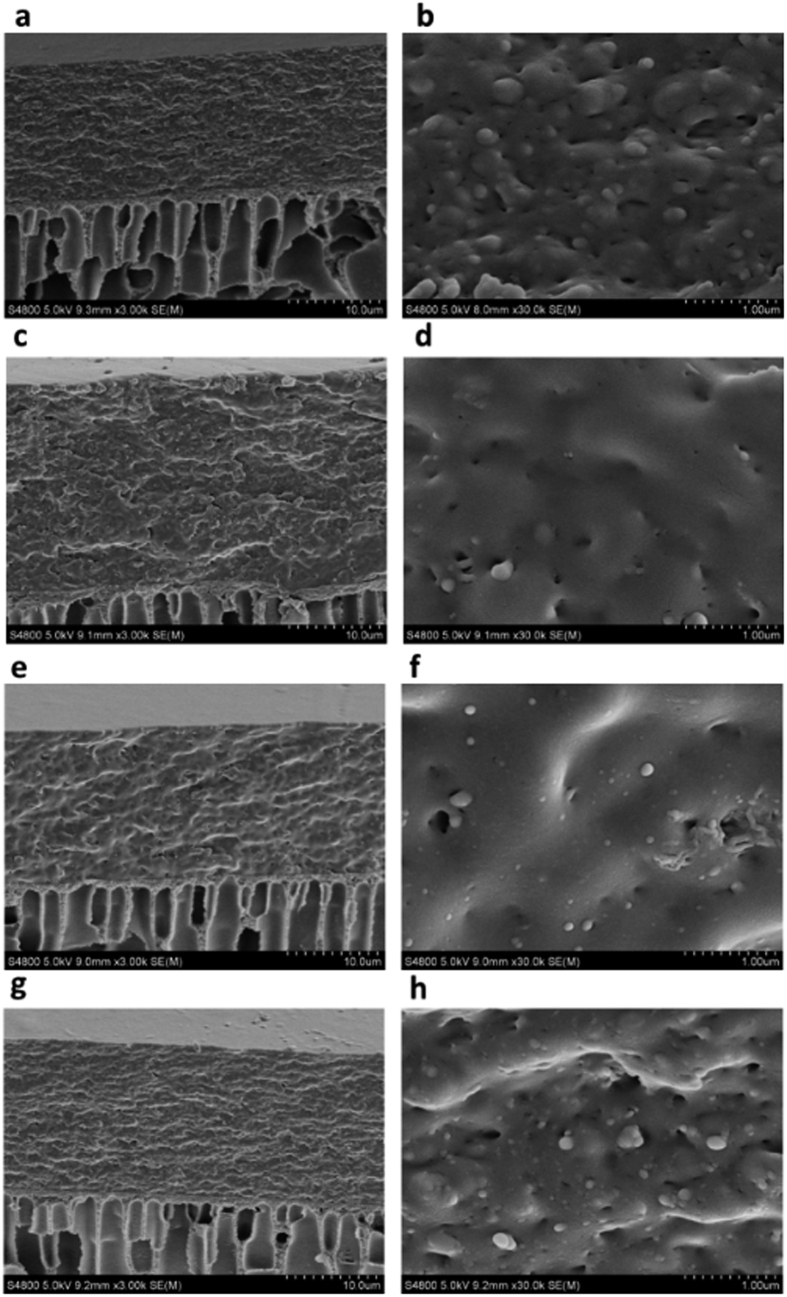
SEM images of cross-section of pure EC membrane (**a,b**), L-rGO MMMs (**c,d**), rGO MMMs (**e,f**) and nanoporous rGO MMMs (**g,h**).

The cross-section morphologies of three MMMs are presented in Fig. 7. It can be observed that the PI ultrafiltration membrane presents a larger finger-like pore structure, which is unfavorable for the permeation of gas. Furthermore, the morphology of membrane has no significant change among EC membrane and MMMs, indicating a superb compatibility between graphene nanosheets and EC polymer^42^,^43^. Magnified images of cross-section morphologies are shown in Fig. 7(b,d,f,h). It is evident that no nanofillers are distinguished and MMMs are relatively flatter than that of EC, which indicates that graphene nanosheets are well dispersed in EC polymer matrix. According to Fig. 6, incorporation of nanofillers into the EC polymer matrix hinders the mobility of macromolecular segments and increases its chains crystallinity, thus graphene nanosheets can act as a nucleating agent in the polymer membranes^44^,^45^,^46^. The excellent dispersion and compatibility was observed, since graphene nanosheets with high aspect ratio can increase the interfacial area between EC polymer chains and nanosheets, resulting in a better compatibility and dispersion in polymer matrix.

**Figure 8:**
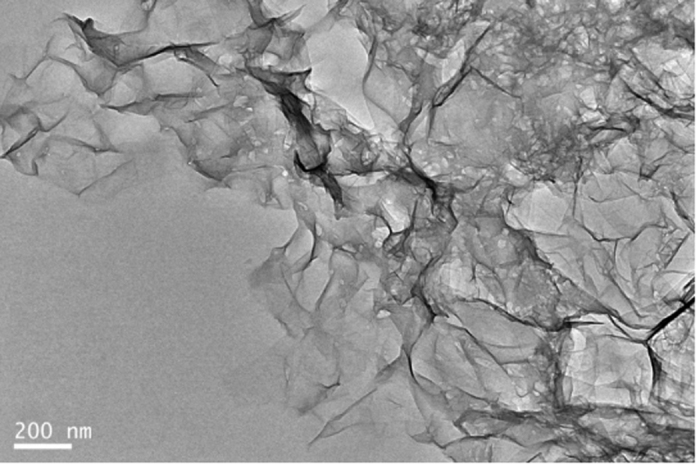
TEM image of nanoporous rGO nanosheets in the MMMs solution.

The TEM image of nanoporous rGO MMMs solution further verifies the nanoscale morphology of nanoporous rGO nanosheets. As observed in Fig. 8, a relatively ambiguous morphology of nanoporous graphene nanosheets was observed in nanoporous rGO in MMMs, probably due to the existence of EC chains. Moreover, it can be clearly seen that there are plenty of nanopores in the nanoporous rGO nanosheets, and these nanopores are facile channels for the small gas molecules to permeate. These phenomena are complementary with the TEM images of nanoporous rGO nanosheets described in the earlier section.

### Membrane permeation performance

**Figure 9:**
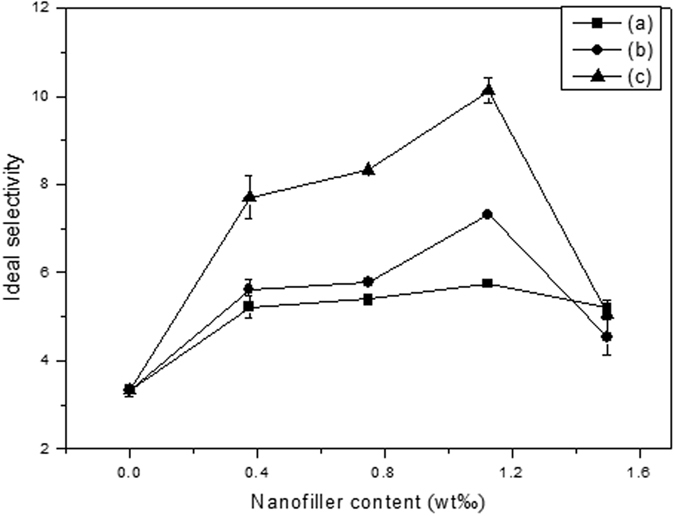
Effect of graphene nanosheets content in the EC polymer matrix on the C_3_H_6_/C_3_H_8_ ideal selectivity: (**a**) L-rGO MMMs; (**b**) rGO MMMs; (**c**) nanoporous rGO MMMs. (Feed pressure at 0.1 MPa, temperature at 298 K).

**Figure 10:**
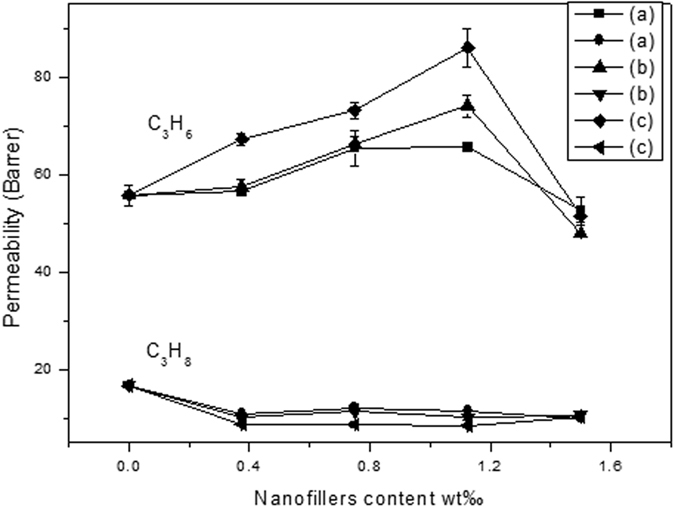
Effect of three graphene nanosheets content in EC polymer matrix on the permeability of C_3_H_6_ and C_3_H_8_: (**a**) L-rGO MMMs; (**b**) rGO MMMs; (**c**) nanoporous rGO MMMs. (Feed pressure at 0.1 MPa, temperature at 298 K).

In order to investigate the structure of graphene nanosheets and their loading content on the membrane permeation performances, three kinds of MMMs are fabricated with different nanofiller loading. Figures 9 and 10 present the correlation between the ideal selectivity and permeability with different loading of graphene nanosheets in the EC polymer matrix. As shown in Figs 9 and 10, C_3_H_6_ permeability, C_3_H_6_/C_3_H_8_ ideal selectivity gradually increase with the increase of graphene nanosheets content from 0 to 1.125 wt‰, while C_3_H_8_ permeability decreases with the content of graphene nanosheets increase from 0 to 0.375 wt‰ and then remain unchanged after 0.0375 wt‰ of graphene nanosheets loading. However, when the loading of the graphene nanosheets in the EC polymer matrix increases to 1.5 wt‰, both C_3_H_6_ permeability and C_3_H_6_/C_3_H_8_ ideal selectivity decrease, indicating that the graphene nanosheets are theoretically impermeable to all atoms and molecules, and greater loading in the MMMs could cause the agglomeration of the graphene nanosheets, resulting in the decrease of the effective surface area^47^,^48^,^49^,^50^. With 1.125 wt‰ of L-rGO, rGO and nanoporous rGO nanosheets loading in the EC polymer matrix, the ideal selectivity of the MMMs could reach as high as 5.74, 7.29 and 10.42 with C_3_H_6_ permeability of 66.05, 76.34 and 89.95 Barrer, respectively. Meanwhile, it is clearly showed that the ideal selectivity of pure EC polymer membrane is 3.45 and the permeability of C_3_H_6_ and C_3_H_8_ is 57.9 and 16.78 Barrer, which is consistence with the related literature^51^. The notable increment involved in both C_3_H_6_ permeability of 1.55-fold and C_3_H_6_/C_3_H_8_ ideal selectivity of 3.02-fold confirm the fact that the nanopores blended into the MMMs enhance the C_3_H_6_ permeability as compared with the pure EC membrane gas permeation performance. However, the blending of graphene nanosheets into the EC polymer matrix hinders the C_3_H_8_ permeability. Among three kinds of MMMs, nanoporous graphene MMMs have C_3_H_6_/C_3_H_8_ ideal selectivity and C_3_H_6_ permeability advantages over that of L-rGO and rGO MMMs, which indicates that oxygen-containing functional groups decrease the C_3_H_6_ permeability. A reasonable explanation of the gas separation mechanism is proposed as shown in Fig. S6, and 1.125 wt‰ of nanofiller loading is chosen as the optimal loading in MMMs for the further discussion of gas permeation properties.

**Figure 11:**
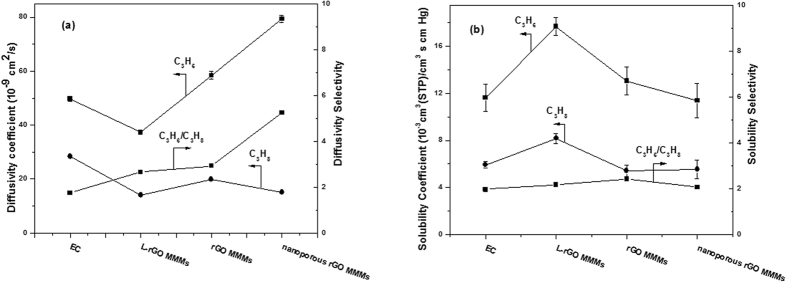
Diffusion coefficient (**a**) and solubility coefficient (**b**) of C_3_H_6_ and C_3_H_8_ in the membranes. (Feed pressure at 0.1 MPa, temperature at 298 K).

To further explore the effect of nanopore in the graphene nanosheets on the gas permeation performance, the diffusivity coefficient (D) and solubility coefficient (S) of the membranes with the optimal 1.125 wt‰ of nanofiller loading are compared in Fig. 11. As shown in Fig. 11(a), it is found that C_3_H_6_/C_3_H_8_ diffusivity selectivity of L-rGO MMMs, rGO MMMs and nanoporous rGO MMMs are higher than that of pure EC membrane. It is notable that the nanoporous rGO MMMs has the highest C_3_H_6_/C_3_H_8_ diffusivity selectivity among L-rGO MMMs and rGO MMMs. The lack of nanopores in the nanosheets hinders the further enhancement of C_3_H_6_ diffusivity coefficient, even L-rGO nanosheets and rGO nanosheets generate a rigid interface with EC polymer chains and increase the tortuous pathway of the gas diffusion to enhance the diffusivity selectivity. We can conclude that the nanopores in the nanosheets further increase the tortuous pathway of the C_3_H_6_ diffusion with less resistance but restrain the diffusion of C_3_H_8_ molecules.

Figure 11(b) shows the gas solubility coefficients and C_3_H_6_/C_3_H_8_ solubility selectivity of L-rGO MMMs, rGO MMMs and nanoporous rGO MMMs with the optimal 1.125 wt‰ of nanofiller loading. The solubility of C_3_H_6_ and C_3_H_8_ in the polymer membranes depends on their relative condensability, characterized by critical temperature, the interaction between polymer and gases, the fraction and amount of free volumes in glassy polymers. The critical temperatures of C_3_H_6_ and C_3_H_8_ are in the following order: C_3_H_6_(364.76 K) < C_3_H_8_(369.8 K). It is believed that higher condensability has the higher solubility of gas in the polymer matrix^52^,^53^,^54^,^55^, however, C_3_H_6_ exhibits greater solubility than C_3_H_8_. This is thanks to the high electron cloud on the double bond with high polarity and flat structure, resulting in more affinity of C_3_H_6_ to generate interaction with polymer, and thus enhancing the C_3_H_6_ solubility. Moreover, the incorporation of graphene nanosheets increases the density of polar groups such as -COOH and -OH in the MMMs. As a result, it creates polar spaces between the interface of nanofillers and polymer. This formation of polar spaces would result in the enhancement in the solubility coefficient of condensable gases^56^.

The diffusivity selectivity increases by 5.24-fold in comparison with 2.66-fold for L-rGO MMMs and 2.93-fold for rGO MMMs, although the loading of nanoporous graphene nanosheets in the polymer matrix cannot efficiently increase the solubility selectivity of the gases due to the decrease of the free volume of the glassy polymer. The incorporation of nanoporous graphene nanosheets into the EC polymer matrix mainly affects the diffusivity selectivity of C_3_H_6_/C_3_H_8_ molecule to enhance the ideal selectivity of MMMs, due to the existence of nanopores and the formation of rigid interface.

In summary, nanoporous rGO nanosheets were successfully fabricated by sodium hydroxide using a facile hydrothermal treatment. The morphology and micro-structure of the nanoporous rGO material were confirmed by TEM and XPS measurement. Nanoporous rGO nanosheets were incorporated into the EC polymer matrix to prepare the MMMs for the C_3_H_6_/C_3_H_8_ permeation. The permselectivity of nanoporous rGO MMMs exhibits a significant increase with the simultaneous enhancement of diffusivity selectivity and solubility selectivity, especially the 5.24-fold increase of the diffusivity selectivity. This result indicates that nanoporous rGO nanosheets are superior in terms of enhancing the diffusivity selectivity of C_3_H_6_ gas molecule. The ideal selectivity for C_3_H_6_/C_3_H_8_ and the permeability coefficient for C_3_H_6_ exhibit a significant increase of 3.02-fold and 1.55-fold in comparison with the pristine EC membrane, respectively. This study shows that nanoporous rGO nanosheets can be effective nanofiller in the polymer matrix to enhance the C_3_H_6_/C_3_H_8_ permeability.

## Methods

### Materials

EC (Mw = 200,000) was purchased from Shanghai Reagent Corporation (China). Graphite oxide was obtained from Nanjing XFNANO Materials Tech Co. Ltd. (China). Sodium borohydride (NaBH_4_), sodium hydroxide (NaOH), sodium dodecylbenzene sulfonates (SDBS), hydrochloric acid (HCl 36~38%), acetone and all other types of reactants were bought from Shanghai Chemical Co., Ltd. (China). The propylene (purity > 99.5%) and propane (purity > 99.9%) were purchased from KODI Gas Chemical Industry Co. Ltd. (Foshan, China).

### Fabrication of LGO, rGO and nanoporous rGO nanosheets

In this case, GO colloidal solution (0.1 g in 150 mL deionized water) was obtained using a mild ultrasonic exfoliation method for 2 h. The above colloidal solution was centrifuged to remove the impurities under 8000 r min^−1^ for 10 min. The rGO and L-rGO nanosheets were obtained through chemical reduction with the mass ratio of GO and NaBH_4_ 1:25 at 80 °C for 2 h and 1:2 at 80 °C for 0.5 h respectively. The reduction solutions were subsequently collected based on filtration, flushing with deionized water and ethanol, and then the resulting products were stored in ethanol at a given concentration (0.012 g in 15 mL ethanol).

The nanoporous rGO nanosheets were synthesized with the treatment of sodium hydroxide under hydrothermal condition. Firstly, GO colloidal solution (0.1 g in 150 mL deionized water) obtained from an mild ultrasonic exfoliation method for 2 h was chemically reduced to L-rGO using the GO to NaBH_4_ mass ratio of 1:2 at 80 °C for 0.5 h, and then the above L-rGO was collected by filtration. Subsequently, 10 mL SDBS (0.086 mol L^−1^) and 20 mL NaOH (12.5 mol L^−1^) were successively added dropwise to form a 60 mL solution. After stirred for 0.5 h, the reaction solution was decanted into a 100 mL Teflon-lined stainless steel autoclave and conducted the hydrothermal treatment at 180 °C for 3 h. Eventually, the reaction solution was cooled to ambient temperature, and the resulting solution was consecutively filtered and washed with sufficient deionized water (five times) and ethanol, and the nanoporous rGO nanosheets were placed in ethanol at a given concentration (0.012 g in 15 mL ethanol).

### Fabrication of MMMs

A series of different concentration of L-rGO, rGO and nanoporous rGO ethanol solutions were prepared via sonication for 1 h. Dried EC (in a vacuum situation at 40 °C for 24 h) was dissolved in methylbenzene to form a solution with the concentration of 19.7 wt%. Under a nitrogen atmosphere and at ambient temperature, the above dispensed graphene ethanol solutions were then added dropwise to the EC solutions with stirring, The resulting casting solution had a mass ratio of graphene to EC of 0.375, 0.75, 1.125 and 1.5 wt‰ respectively. After 24 h dissolution, the casting solutions were filtered with stainless steel filter, and still degassing for 24 h. The above casting solution was then cast onto the polyimide (PI) ultrafiltration supported membrane using a micrometer film applicator under RH 40% at 298 K. The final membranes were subsequently preserved for gas separation tests. All membrane thicknesses were approximately 15–20 μm.

## Additional Information

**How to cite this article**: Yuan, B. *et al*. Propylene/propane permeation properties of ethyl cellulose (EC) mixed matrix membranes fabricated by incorporation of nanoporous graphene nanosheets. *Sci. Rep.* **6**, 28509; doi: 10.1038/srep28509 (2016).

## Supplementary Material

Supplementary Information
