# Supplementary material for: Propylene/propane permeation properties of ethyl cellulose (EC) mixed matrix membranes fabricated by incorporation of nanoporous graphene nanosheets
Source: Sci Rep. 2016 Jun 29;6:28509. doi: 10.1038/srep28509 (PMC4926224; doi:10.1038/srep28509)
Supplement: Supplementary Information [file srep28509-s1.pdf]

Supplementary Information

Propylene/propane permeation properties of ethyl cellulose (EC) mixed matrix membranes fabricated by incorporation of nanoporous graphene nanosheets

Bingbing Yuan[a]†, Haixiang Sun[a,b]*†, Tao Wang[b], Yanyan Xu[b], Peng Li[a], Ying Kong[a], Q. Jason Niu[a]*

[a] *State Key Laboratory of Heavy Oil Processing, China University of Petroleum (East China), Qingdao 266580, P.R. China*

[b] *College of Science, China University of Petroleum (East China), Qingdao 266580, P.R. China*

†Contributed equally to this paper and share first authorship.

*Corresponding authors.

Tel.: +86 532 86981135; fax: +86 532 86981135

E-mail address: sunhaixiang@upc.edu.cn (Dr. Sun)

qjniu@upc.edu.cn (Dr. Niu)

Characterization

The samples for transmission electron microscopy (TEM) analysis were prepared by a small amount of L-rGO, rGO and nanoporous rGO nanosheets dispersed in ethanol and ultrasonic for 15 min to form a homogeneous suspension. A drop of the suspension was then deposited on a carbon-coated copper grid for TEM observation (JEM 2100F, JEOL, Japan) to get an optical image of graphene nanosheets. Similarly, the nanoporous rGO MMMs casting solution sample for TEM characterization was prepared using 50 ml ethanol to dilute the 0.5 g casting solution with 1.125 wt‰ nanoporous rGO nanosheets, and then was sonicated for 15 min. Subsequently, a drop of suspension was deposited on the copper grid.

Cross-section morphology of EC and graphene nanosheets MMMs was characterized by scanning electron microscopy (SEM, Model S-4800, Hitachi, Japan) after being coated with a conductive layer of sputtered gold. The cross-section was obtained by snap freezing the membranes in liquid nitrogen.

Fourier transform infrared (FTIR) spectroscopy was performed on a Nicolet 6700 to characterize the morphological changes of graphene material. The measured wavenumber range was recorded between 4000 and 400 cm^{-1} at a resolution of 0.4822 cm^{-1}. All original spectra were baseline corrected using the Omnic 6.1 software.

The X-ray photoelectron spectroscopy (XPS) analyses of L-rGO, rGO and nanoporous rGO nanosheets were performed with Kratos Axis Ultra HSA spectrometer. The analysis was carried out with a monochromatic Al Ka X-ray source

(1486.7 eV), operating at 15 kV (90 W), in FAT mode (fixed analyzer transmission), with a pass energy of 40 eV for regions ROI and 80 eV for survey. Data acquisition was performed with a pressure lower than 1×10^{-6} Pa, and a charge neutralization system was used. Survey and multi-region spectra were recorded at C_{1s} and O_{1s} photoelectron peaks. The spectra obtained after a Shirley background subtraction were fitted using XPS PeakFit V4.12 software.

The Raman spectra of L-rGO, rGO and nanoporous rGO nanosheets were obtained in backscattering configuration on a Renishaw inVia Reflex microscope using an Ar+ ion laser (λ = 514.5 nm) and a high power near infrared (NIR) diode laser (λ = 785 nm) as excitation sources. The laser beam was focused onto the samples by means of a 20× objective, while the laser power density was kept below 0.05 mW μm^{-2} to avoid local heating.

X-ray diffraction (XRD) patterns analysis for the graphene nanosheets and MMMs was performed at the application laboratory of PANalytical in Almelo (The Netherlands) with an X'Pert PRO MPD diffractometer in transmission geometry with $\theta/2\theta$ goniometer, using Cu Ka radiation (k = 1.5418 Å) and a focusing X-ray mirror. Each pattern was collected from 5 to 60 (2θ) in repetitive mode (three times) with a total duration of approximately 0.4 h at selected times of hydration.

Thermal Gravimetric Analysis (TGA) experiments were carried out in an aluminum pan using a TGA 6300 thermo gravimetric analyzer at a heating rate of 10 °C min^{-1}, and the nitrogen flux was 40 mL min^{-1}.

A mechanical property testing on EC and MMMs were performed with a TY tester (Model TY8000-A10KN China). The films around an average thickness of 0.150 mm were cut into dumbbell shape with a length of 50 mm and a middle width of 4 mm, and were then clipped with two clamps on the instrument. Afterwards, the testing was performed with a 10 kgf load, 100 mm min^{-1} and 18 mm grip separation to get tensile strength and elongation at break.

BET Characterization

The N_2 adsorption and desorption isotherms were measured at 77.15 K on a TriStar II 3020 V1.03. Prior to the gas sorption measurements, all the samples were outgassed in a vacuum at 200 °C for 24 h. The specific surface area and the pore size distribution were calculated using the Braunauer_Emmett_Teller (BET) method, and the relative pressure range of P/P_0 from 0.1 to 0.3 was used for multipoint BET calculations.

The porosity of prepared graphene nanosheets was analyzed by N_2 gas adsorption-desorption measurements, and Fig. S1 exhibits a characteristic type-IV isotherm with a pronounced hysteresis in the P/P_0 range 0.4-1.0, implying the presence of a large number of micropores and mesopores in the as-prepared nanoporous rGO nanosheets[1]. According to the Brunauer-Emmett-Teller (BET) characterization, the surface area of the nanoporous rGO nanosheets is 322.0058 m^2 g^{-1} (calculated in the linear relative pressure range from 0.1 to 0.3), and the micropore area is 101.6825 m^2 g^{-1}, indicating that the microporous porosity is as high as 31.37%.

Besides, the adsorption average width is about 4.98343 nm. Fig. S2 displays the results of the cumulative pore volume and pore size analysis N_2 adsorption, assuming a slit pore geometry for the micropores and a cylindrical-pore geometry for the mesopores, which appears (although oversimplified) to be a reasonable assumption also with regard to the TEM (see Figure.1) results[2].

Thus, through the BET analysis, it is indicated that the surface of nanoporous rGO nanosheets exhibits large amounts of nanopores.

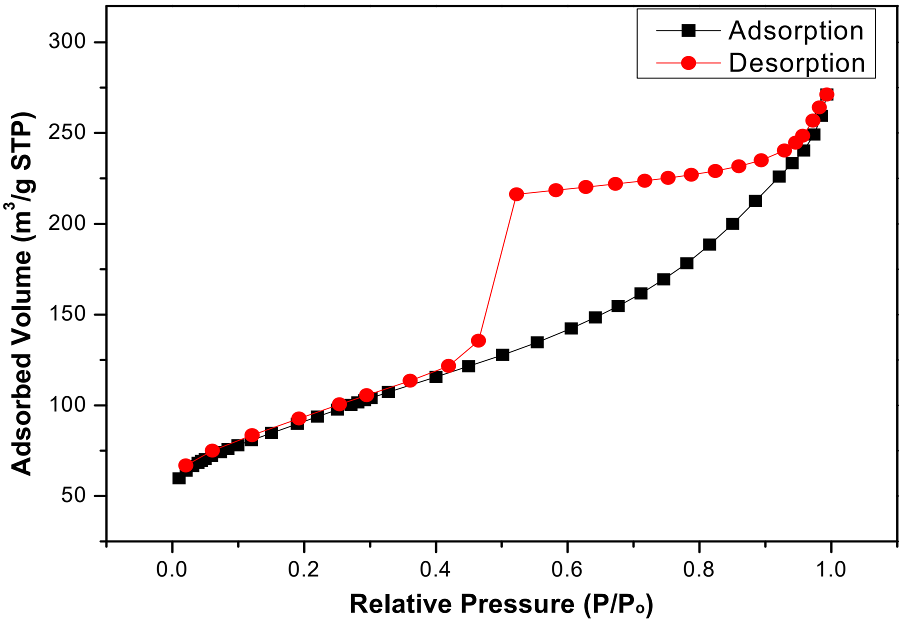

Fig. S1 N_2 sorption isotherms (77 K) on nanoporous rGO nanosheets.

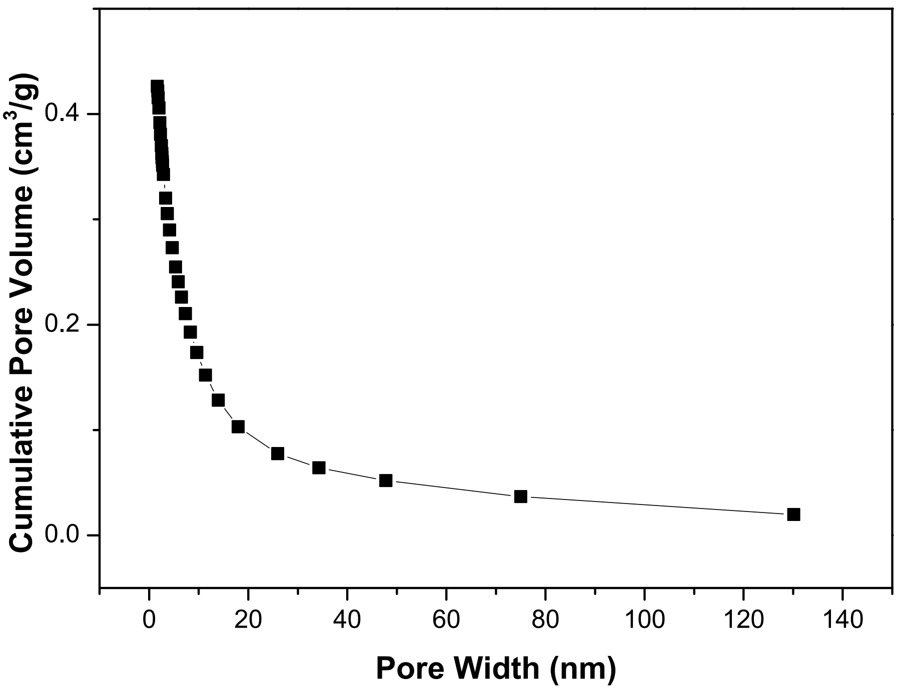

Fig. S2 Cumulative pore volume and pore-size distribution for N_2.

Separation experiment

In this work, the gas permeability of the MMMs was studied by the constant volume variable pressure. To be specific, the permeability performance of membrane for propylene and propane were measured by our previous work[3]. The experiments were performed with pure gases (C_3H_6 and C_3H_8) at the temperature of 298 K respectively, and the effective permeation area of the membrane was 4.95 cm^2. The feed and the permeation cell were evacuated using a vacuum pump for more than 6 h to maintain the degree of vacuum to less than 20 Pa, and then subsequently the feed gas was introduced into the membrane module at a constant pressure provided rate. After the pressure difference between the permeation side and the feed side reached a given digit such as 0.1 MPa, the feed shave was closed and the test process began.

When the pressure change of the permeation side remained constant over time, the test was stopped, and the gas permeation parameter (P) of C_3H_6 and C_3H_8 was obtained using Eq. (1).

$$P = \frac{273\ \ VL\,d_p}{76\ ATp_o d_t} \qquad (1)$$

where P is the permeability of a membrane to a gas and its unit is the Barrer (1 Barrer = 1×10^{-10} $cm^3\cdot cm\cdot cm^{-2}\cdot s^{-1}\cdot cmHg^{-1}$); V is the volume of the down-stream chamber (cm^3); L and A are the thickness (cm) and the effective area of the membrane (cm^2) respectively; T is the experimental temperature (K) and the pressure of the feed gas in the up-stream chamber is given by p_0 in cm Hg.

The diffusion coefficient of gas penetrants in one dimension is given by[4]:

$$D = \frac{L^2}{6\theta} \qquad (2)$$

Where D is the diffusion coefficient ($cm^2\ s^{-1}$); L is the thickness of the activated layer (cm); θ is the initial stage permeation process observed gas diffusion lag time.

Once D and P were obtained, the apparent solubility coefficient, S, could be calculated by Eq. (3). The unit of S is cm^3 (STP) $cm^{-3}cm\ Hg^{-1}$.

$$S = \frac{P}{D} \qquad (3)$$

The ideal separation factor (α) is defined as:

$$\alpha_{A/B} = \frac{P_A}{P_B} \quad (4)$$

Here, P_A and P_B are the permeability coefficients of C_3H_6 and C_3H_8, respectively.

Tensile property and thermal stability of MMMs

Table S1 Tensile property of EC and graphene MMMs

Membrane	S (kN/mm^2)[a]	ΔL(%)[b]
EC	35.26±0.28	2.85±0.15
L-rGO MMMs	40.60±0.57	7.39±0.39
rGO MMMs	38.53±2.42	6.86±0.36
nanoporous rGO MMMs	40.20±0.42	7.13±0.38

[a] Tensile strength. [b] Elongation at break.

The tensile strength and elongation at break of EC membrane and L-rGO MMMs, rGO MMMs and nanoporous rGO MMMs are listed in Table S1. After incorporating graphene nanosheets into EC polymer chains, both tensile strength and elongation at break substantially increase. In essence, graphene MMMs have more excellent mechanical properties than that of pure EC membrane.

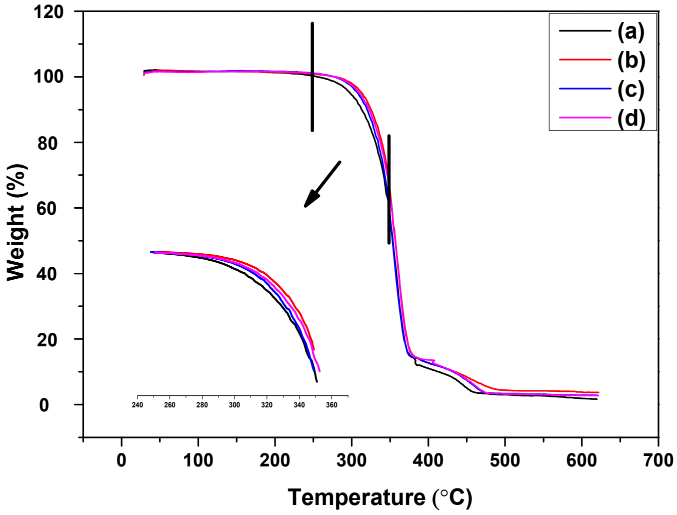

Fig. S3 TGA curves of pure EC membrane (a), L-rGO MMMs (b), rGO MMMs (c), nanoporous rGO MMMs (d).

Thermal stability is another aspect to illustrate whether graphene has an effect on the properties of MMMs. The thermal properties of EC and graphene MMMs with the loading mass ratio of 1.125 wt‰ are analyzed with the TGA instrument. As shown in Fig. S3, EC and graphene MMMs present a similar two-step decomposition process. However, it is clearly observed that MMMs possess a higher decomposition temperature than that of pure EC. Further observation reveals that the average first-stage temperature of degeneration for MMMs is approximate 6 °C higher than pure EC membrane, indicating an increased thermal stability. Meanwhile, the second-stage process also shows a relative increase in degeneration temperature in comparison with pristine polymer membrane.

Feed pressure dependence of nanoporous rGO MMMs

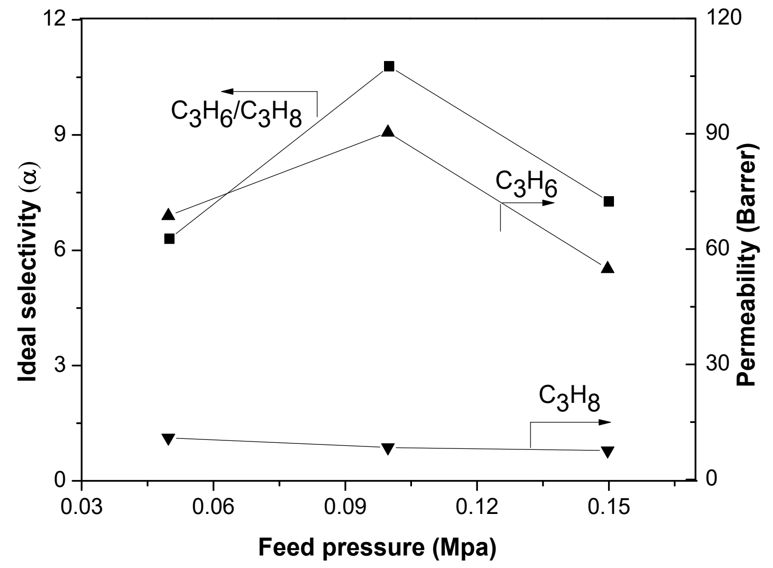

Fig. S4 The effect of feed pressure on C_3H_6/C_3H_8 ideal selectivity and permeability coefficient of nanoporous rGO nanosheets with the mass ratio of 1.125 wt ‰. (Temperature at 298 K)

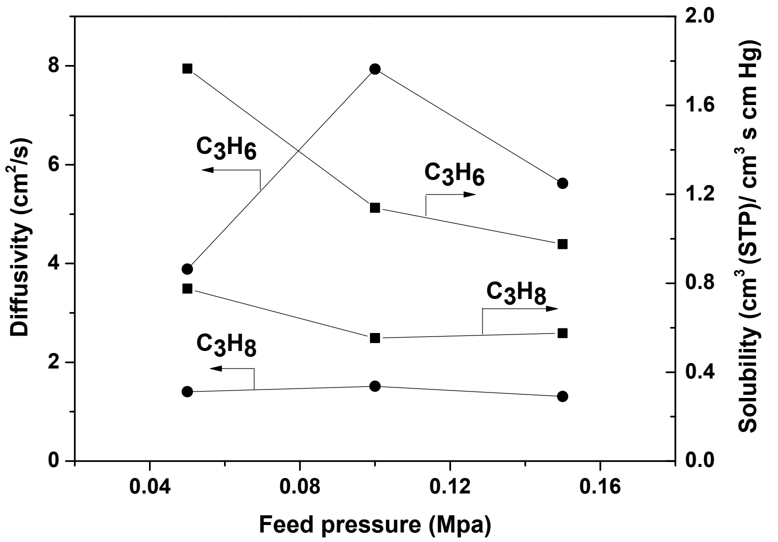

Fig. S5 The effect of feed pressure on solubility coefficient and diffusivity coefficient of C_3H_6 and C_3H_8 of nanoporous rGO MMMs with the mass ratio of 1.125 wt‰. (Temperature at 298 K)

The effect of feed pressure on ideal selectivity, permeability coefficient, solubility coefficient and diffusion coefficient of C_3H_6 and C_3H_8 is given in Fig.

S4 and Fig. S5, respectively. As observed, the ideal selectivity of C_3H_6/C_3H_8 and permeability of C_3H_6 firstly increases and later deceases with the increase of the pressure, whereas the permeability of C_3H_8 declines. Furthermore, the diffusion coefficient of C_3H_6 and C_3H_8 originally rises and subsequently descends, and then the solubility coefficient of C_3H_6 and C_3H_8 decreases. The permeation behavior of gases through the glassy polymer membrane follows the dual model sorption. According to such model, in the low pressure gas easily performs sorption in empty spaces with the free volume in polymer (Langmuir's region), whereas in the high pressure the sorption in dense media of the polymer (Henry's region) plays an important role due to the saturation of Langmuir's region. Therefore, the solubility coefficients of C_3H_6 and C_3H_8 decrease with the increase of the feed pressure. In general, based on partial immobilization theory expressed by Koros and Paul[5], the gas molecules absorbed in Henry's region possess higher diffusion coefficient than that of absorbed in Langmuir's regions. As nanoporous rGO MMMs has a decreasing Langmuir's region characterized by the XRD graph, when the feed pressure increases from 0.05MPa to 0.1MPa, absorption behavior mainly performs in Henry's regions, resulting in a higher diffusion coefficient. However, the reduction on the diffusion coefficient of C_3H_6 and C_3H_8 from 0.1 MPa to 0.15MPa is ascribed to the compaction of polymer chains to decrease the length of the tortuous pathway of the gas diffusion formed by the nanopores.

Among three kinds of MMMs, nanoporous graphene MMMs have C_3H_6/C_3H_8 ideal selectivity and C_3H_6 permeability advantages than that of L-rGO and rGO MMMs,

which indicates that oxygen-containing functional groups decrease the C_3H_6 permeability. A reasonable explanation of the gas separation mechanism is proposed as in Fig. S6. Fig. S6 shows the diffusion pathway of gas molecule in the membranes. As researchers[6-8] indicated, the interfacial void was created between the polymer and the graphene nanosheets, which contributed to the diffusion of gas molecules. In the pristine EC membrane, the diffusion pathway was irregular and friction due to the mobility of polymer matrix chains and the existence of oxygen-containing groups in the EC polymer. In the MMMs, with the generation of interfacial void in the rigidified interface, gas molecule was prone to diffuse. Especially for the MMMs (nanoporous rGO nanosheets), the gas molecule diffusion pathway was strongly further optimized due to the existence of nanopores, which was regular, small friction and shortcut diffusion.

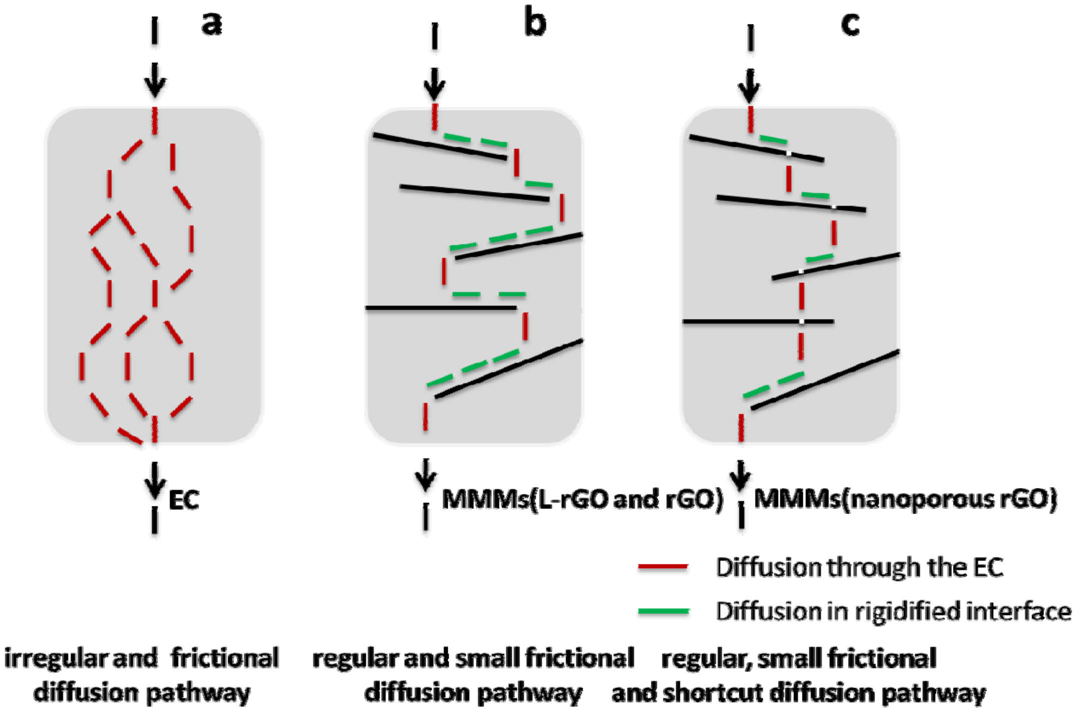

Fig. S6 The diffusion pathway of gas molecule in the membranes

Reference

1. Kim, T. Y., Jung, G., Yoo, S., Suh, K. S., Ruoff, R. S. Activated graphene-based carbons as supercapacitor electrodes with macro- and mesopores. *ACS. Nano.* **7**, 6899–6905 (2013).
2. Zhu, Y. W. *et al.* Carbon-based supercapacitors produced by activation of graphene. *Science* **332**, 1537 (2011).
3. Sun, H. *et al.* Preparation and characterization of C_{60}-filled ethyl cellulose mixed-matrix membranes for gas separation of propylene/propane. *Chem. Eng. Technol.* **37,** 611–619 (2014).
4. Xu, J. W., Chng, M. L., Chung, T. S., He, C. B., Wang, R. Permeability of polyimides derived from non-coplanar diamines and 4,40-(hexafluoro-isopropylidene) diphthalic anhydride. *Polymer* **44,** 4715–4721 (2003).
5. Paul, D. R., Koros, W. J. Effect of partially immobilizing sorption on permeability and the diffusion time lag. *J. Polym. Sci. B, Polym. Phys.* **14,** 675–685 (1976).
6. Cong, H., Hu, X., Radosz, M., Shen, Y. Brominated poly (2,6-diphenyl-1,4-phenylene oxide) and its silica nanocomposite membranes for gas separation. *Ind. Eng. Chem. Res.* **46,** 2567−2575 (2007).
7. Cao, K., *et al.* Enhanced water permeation through sodium alginate membranes by incorporating graphene oxides. *J. Membr. Sci.* **469,** 272−283 (2014).
8. Mahajan, R., Koros, W. J. Mixed matrix membrane materials with glassy polymers. Part 1. *Polym. Eng. Sci.* **42,** 1420−1431 (2002).
